# Role of Viral Protein U (Vpu) in HIV-1 Infection and Pathogenesis

**DOI:** 10.3390/v13081466

**Published:** 2021-07-27

**Authors:** Nabab Khan, Jonathan D. Geiger

**Affiliations:** Department of Biomedical Sciences, School of Medicine and Health Sciences, University of North Dakota, 504 Hamline Street, Room 110, Grand Forks, ND 58203, USA; nabab.khan@ndus.edu

**Keywords:** human immunodeficiency virus-1 and -2, simian immunodeficiency viruses, viral protein U (Vpu), bone marrow stromal antigen 2, transmembrane domain, endoplasmic reticulum-associated degradation pathway, endosomal sorting complexes required for transport, endolysosomes, autophagy

## Abstract

Human immunodeficiency virus (HIV)-1 and HIV-2 originated from cross-species transmission of simian immunodeficiency viruses (SIVs). Most of these transfers resulted in limited spread of these viruses to humans. However, one transmission event involving SIVcpz from chimpanzees gave rise to group M HIV-1, with M being the principal strain of HIV-1 responsible for the AIDS pandemic. Vpu is an HIV-1 accessory protein generated from *Env*/*Vpu* encoded bicistronic mRNA and localized in cytosolic and membrane regions of cells capable of being infected by HIV-1 and that regulate HIV-1 infection and transmission by downregulating BST-2, CD4 proteins levels, and immune evasion. This review will focus of critical aspects of Vpu including its zoonosis, the adaptive hurdles to cross-species transmission, and future perspectives and broad implications of Vpu in HIV-1 infection and dissemination.

## 1. Introduction

Human immunodeficiency virus (HIV) is a lentivirus that belongs to the Retroviridae family. The HIV retrovirus contains two RNA molecules with three prototypic genes that encode group-specific antigen (*gag*), envelope (*env*), and polymerase (*pol*) proteins [1,2,3]. HIV isolates have been classified into two types: HIV-type 1 (HIV-1) and HIV-type 2 (HIV-2). HIV-1 is the causative agent of HIV/AIDS, while HIV-2 is constrained to some well-defined Central and Western Africa regions; relative to HIV-1, HIV-2 has weak transmission capabilities [4,5]. Based on phylogenetic analysis, HIV-1 shows substantial similarities to the virus SIVgor that infects gorillas (Gorilla gorilla) and the virus SIVcpz that infects chimpanzees (Pan troglodytes troglodytes). HIV-2 resembles the virus SIVsmm that infects sooty mangabey monkeys (Cercocebus atys). HIV-1 evolved from chimpanzees and/or gorillas by independent cross-species transmissions of SIVs [4,5].

The HIV-1 virus has been subdivided into M, N, O, and P subtypes according to their origin and distribution patterns within the human population. HIV-1 M is globally distributed and is a major factor causing the pandemic disease AIDS. HIV-1 N is rare (non-major/non-outlier) and originated from chimpanzees [5]. HIV-1 O and P groups have close relationships to the virus SIVgor isolated from gorillas; geographically, the O group virus is constrained to Cameroon and surrounding countries [5]. The P group virus was discovered to come from two individuals in Cameroon. Vpu protein is encoded by all groups of HIV-1, but biological differences have been noted between the various sources of Vpu proteins. Pandemic M group viruses contain Vpu proteins that are more highly active than do other groups [5]. Clearly, one needs to understand the functions of Vpu proteins to understand their roles in controlling HIV-1 infection and HIV/AIDS disease pathogenesis.

## 2. *Vpu* Gene and Its Diversification

Vpu was initially characterized as an U open reading frame (ORF) product localized in the HIV-1 genome between the *env* and *tat* exons [6]. The Vpu protein is translated from bicistronic mRNA of env-vpu presumably through leaky scanning of ribosomes from the initiation codon of the *vpu* gene [7,8]. The *vpu* gene is encoded in the HIV-1 genome, but it is not present in the genomes of HIV-2 and of SIVs such as SIV from rhesus macaques (SIVmac) and SIV from sooty mangabey (SIVsmm) [4,5,9]. However, structural homologs of Vpu have been identified in SIV from chimpanzee (SIVcpz), as well as in SIV from the greater spot-nosed monkey (Cercopithecus nictitans; SIVgsn), the mona monkey (Cercopithecus mona; SIVmon), the mustached monkey (Cervicopithecus Cephus; SIVmus), Dent’s mona monkey (Cercopithecus mona denti; SIVdent), and recently in gorilla (Gorilla gorilla; SIVgor) [9,10,11,12,13].

## 3. Vpu Protein and Its Cellular Distribution

**Figure 1:**
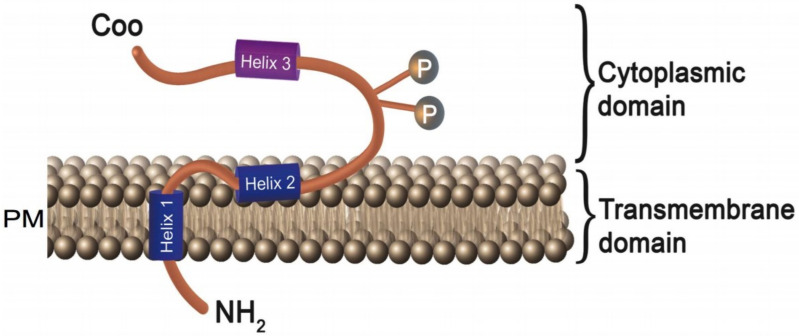
Structure of the human immunodeficiency virus (HIV)-1 accessory protein Vpu. Vpu protein is composed of three different distinct alpha helices: the N-terminus proximal transmembrane domain (Helix1-TMD: 6–29 residues) and a cytoplasmic domain that consists of two alpha helices (Helix 2: 32–52 residues, Helix 3: 57–72 residues). The first cytoplasmic helix shows amphipathic behavior with hydrophobic and polar residues on the sides. The hydrophobic portion is buried in the cell membrane, while the hydrophilic region is exposed to the cytoplasmic side. The second cytoplasmic helix is formed by acidic amino acids. Two phosphorylated serine residues, S52 and S56, interconnect these cytoplasmic helices.

Vpu is a multimeric integral membrane phosphoprotein with 81 amino acids [14,15]. It has three distinct alpha-helices: the N-terminus proximal transmembrane domain (Helix1-TMD: 6–29 residues) and two C-terminus domains, Helix 2 (32–52 residues) and Helix 3 (57–72 residues) [16,17,18,19] (Figure 1). Helix-2 is amphipathic and is hydrophobic with polar residues on the sides. The hydrophobic portion is buried in plasma membranes, while the hydrophilic region is cytoplasmic [20]. Helix-3 contains acidic amino acids interconnected by two phosphorylated Serine residues: S52 and S56 [21]. Protein kinase casein kinase 2 (CK-2) catalyzes the phosphorylation of the serines (Figure 1) and these post-translational modifications regulate associations between Helix 3 and beta-TrCP/ubiquitin ligase complexes [22,23]. Vpu once oligomerized can form pentameric pore-like structures through which selective monovalent cations can pass [24,25,26].

Vpu proteins localize to the plasma membranes, endoplasmic reticulum (ER), and trans-Golgi network (TGN) [27,28,29,30]. Sequence analysis of the cytosolic domain of Vpu shows the presence of putative trafficking signals that carry variations in amino acid residues among different subtypes of Vpu. These signals include YXXΦ, a conserved tyrosine-based sorting motif (where Φ represents a hydrophobic residue), and a ([D/E] XXXL [/I/V]) sorting motif consisting of acidic residue/dileucine-based sequences that are present in the hinge portion between the cytosolic domain and the TMD. The latter is involved in endocytosis and the targeting of transmembrane host proteins to lysosomes [31]. Another ([D/E] XXXL [/I/V]) motif is present in the second alpha-helix of the cytoplasmic domain [29]. Several primary isolates of HIV-1 and laboratory-adapted viruses carry polymorphisms of the *vpu* gene that are based on variations of putative trafficking signal sequences [32] and these polymorphisms regulate subcellular distribution patterns and biological activities of the Vpu protein.

## 4. Role of Vpu Protein in HIV-1 Pathogenesis

Vpu has two well-established functions in HIV-1 infection. First, through the ubiquitin-proteasomal pathway, it enhances the degradation of CD4 protein produced de novo in the endoplasmic reticulum [33]. Second, Vpu augments the release of progeny virions from infected cells [23,34,35] by counteracting the effect of Tetherin, a host restriction factor. Tetherin, also known as BST-2, CD317, or HM1.24, strongly inhibits the release of virions from infected host cells [28,36,37]. In addition, Vpu also regulates the transport of host proteins from ER to Golgi [38], modulates MHC class II presentation [39], induces the stabilization of p53 [40], induces degranulation of natural killer cells (NK cells) by NTBA downmodulation, inhibits lipid antigen presentation through CD1d downmodulation [41,42], induces apoptosis, and impairs migration and chemotactic signaling within CD4^+^ T-cells through CCR7 downregulation [43].

### 4.1. Role of Vpu in HIV-1-Induced CD4 Receptor Downregulation

CD4 is the primary receptor through which primate lentiviruses enter target cells [44]. It is a 54 kDa type-I integral glycoprotein expressed on the surface of cells including helper T-lymphocytes, monocyte/macrophage lineage cells, and hematopoietic progenitor cells. HIV-1 infection of these cells leads to a reduction in the cell surface levels of CD4 receptors [45]. CD4 downregulation has been proposed to block the superinfection of target cells [46] and protect the infected cell from host immune responses, and favors viral replication fitness [47] (Table 1). Constitutive expression of CD4 can be harmful to productive viral replication and dissemination [48]. De novo produced CD4 molecules bind Env polyproteins with high affinity within the endoplasmic reticulum, prevent the transport and processing of Env precursor to its products gp41 and gp120 [49,50], and reduce secretion of infectious progeny virion particles from infected cells.

**Figure 2:**
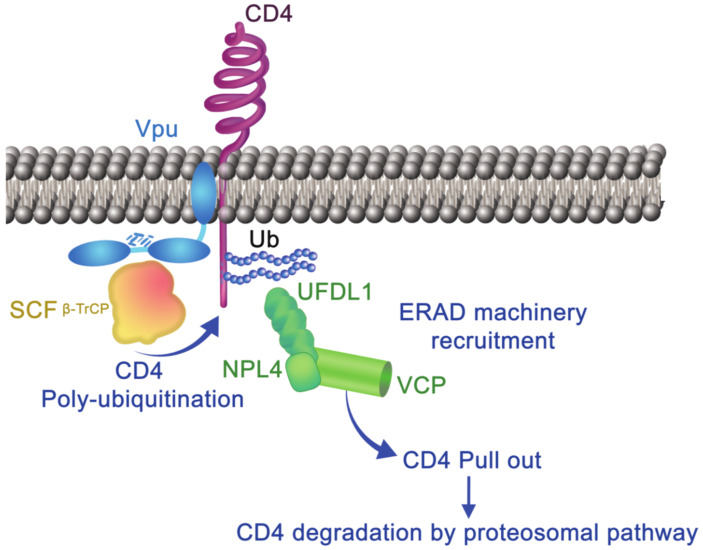
Endoplasmic reticulum-associated degradation of CD4 by Vpu protein: Vpu interacts with CD4 by transmembrane–transmembrane domain in the ER and promotes binding with SKP1-cullin1-F-Box (SCF) E3 ubiquitin ligase through the SCF subunits β-TrCP1 and β-TrCP2. Vpu and SCF β-TrCP complex induces proteasomal degradation of CD4 by the ERAD pathway by extracting it from ER. Abbreviations: Vpu: viral protein U, CD4: cluster differentiation 4, Ub: ubiquitination, SCF β-TrCP: SKP1-cullin1-F-Box-β-transducin repeat-containing proteins, UFDL1: ubiquitin fusion degradation 1-like, NPL4: nuclear protein localization protein 4, ERAD: endoplasmic reticulum-associated degradation, VCP: valosin-containing protein.

Vpu can enhance degradation of de novo produced CD4 by retention of CD4 in the endoplasmic reticulum through interactions between Vpu and CD4 via their transmembrane domains, poly-ubiquitination of CD4, and transport to the ERAD (ER-associated degradation) pathway [33,57,58]. The integrity of the cytoplasmic domain of Vpu protein and the DSGXXS motif containing the S52/S56 phosphoserine residues are critical to proteasomal degradation of CD4; Vpu interacts directly with β-TrCP1, β-TrCP2 (β-transducin repeat-containing protein 1 or 2), two adaptor molecules (SKP1-cullin-F-Box), and the E3 ubiquitin ligase complex [59,60]. The interaction of SCF- β-TrCP with Vpu induces the poly-ubiquitination of CD4 through binding to lysine, serine, and threonine residues [58] (Figure 2). Without those residues, SCF-mediated interactions between Vpu with CD4 result in retention of the receptor in the ER. Without the ERAD complex (VCP-UFDIL-NPL4), Vpu fails to pull CD4 out of the endoplasmic reticulum and reduces proteasomal degradation [58] (Figure 2). Decreased interactions between β-TrCP/Vpu reduce proteasomal degradation by the ERAD pathway of several cellular factors including β-catenin, ATF4, and p53 [40].

### 4.2. Role of Vpu in BST-2 Downregulation and Virus Release

Vpu increases the release of HIV-1 from HIV-1-infected cells [15,34]. As confirmed by electron microscopy (EM), Vpu defective virion particles accumulate at the budding site on infected host cell membranes [35,61]. The role of Vpu in virus release varies between cell types. For example, the release of Vpu-defective HIV-1 particles was strongly reduced in HeLa cells, but it was not affected in COS, HEK293T, CV-1, and Vero cells [62,63,64]. The BST-2 protein was identified as a restriction factor that blocked the release of mutant viruses lacking the accessory gene *vpu* [28,36] that is constitutively expressed in HeLa cells, but not in permissive cell lines like HT1080 and HEK293T. The expression of BST-2 and its restrictive phenotype could only be maintained by IFN-α/β induction in permissive cells and increased in Jurkat and primary CD4^+^ T-cells. Moreover, the induction of BST-2 expression in HT1080 and HEK293T cells restricted the secretion of virus particles in the absence of Vpu protein [28,36] and siRNA-mediated reduction of BST-2 expression in HeLa cells led to the efficient release of Vpu defective virion particles [28,36].

BST-2 can inhibit the release of almost all enveloped viruses, including retroviruses, flaviviruses, herpesviruses, arenaviruses, rhabdoviruses, and paramyxoviruses [65,66]. Therefore, BST-2 has been proven to be a critical innate immune factor for restricting viral release. Primate lentiviruses express three different proteins that counteract BST-2 antiviral activity: Vpu for HIV-1 [28,36]; Nef for SIV major isolates [51,67,68]; and Env for HIV-2, SIVagmTan, and SIVmac239Δnef isolates [69,70,71,72]. HIV-1 Vpu, HIV-2 Env, and SIV Nef all decrease cell surface levels of BST-2 from budding viral sites to favor virus release [69,70,71,72].

BST-2 is a 30–36 kDa type II integral membrane protein that is expressed constitutively as well as following induction by type-I interferon or other pro-inflammatory signals [65]. It consists of a short N-terminal cytoplasmic tail inter-linked to a transmembrane domain and an extracellular domain anchored in the membrane via its glycosylphosphatidylinositol (GPI) moiety in the C-terminal region [73]. BST-2 is distributed mainly in cholesterol-rich microdomains of the cell membrane and intracellular compartments such as the trans-Golgi-network (TGN) and endosomes [73,74].

BST-2 can physically tether de novo generated virion particles at the cell membrane of infected cells, thereby decreasing virus release [75,76]. This tethering occurs following formation of homodimers via parallel disulfide-bonding and cross-linking with virions particles and plasma membranes through its membrane anchoring N-terminal domains [75,77]. BST-2 makes “axial” arrangements in which the BST-2 GPI anchors remain connected to the membrane of infected cells. Vpu downregulates BST-2 and interactions between Vpu and BST-2 utilize their respective transmembrane domains. The Ala14, Ala18, and Trp22 residues of the Vpu TMD are crucial for BST-2 downregulation from the cell membrane through direct interaction with specific residues on BST-2 (Val, Iso, Leu, and Leu) [78,79]. These residues are involved in TMD–TMD interactions, create an anti-parallel helix–helix interface [80], and maintain the interaction between these proteins [81,82]. The antagonistic effect of Vpu on BST-2 activity takes place by three sequential steps: downregulation from the cell surface, restriction of BST-2 recycling, and decline in intracellular BST-2 levels. Downregulation of BST-2 at the cell surface is mediated by clathrin-coated vesicles through direct interaction of the AP2 (clathrin adaptor complex) with a Y6XY8 motif (non-canonical dual Tyrosine residues) present in the cytoplasmic tail of BST-2 [74,83]. Vpu restricts the recycling of internalized BST-2 to the cell membrane and blocks the translocation of de novo generated BST-2 to the cell membrane [84,85,86,87].

**Figure 3:**
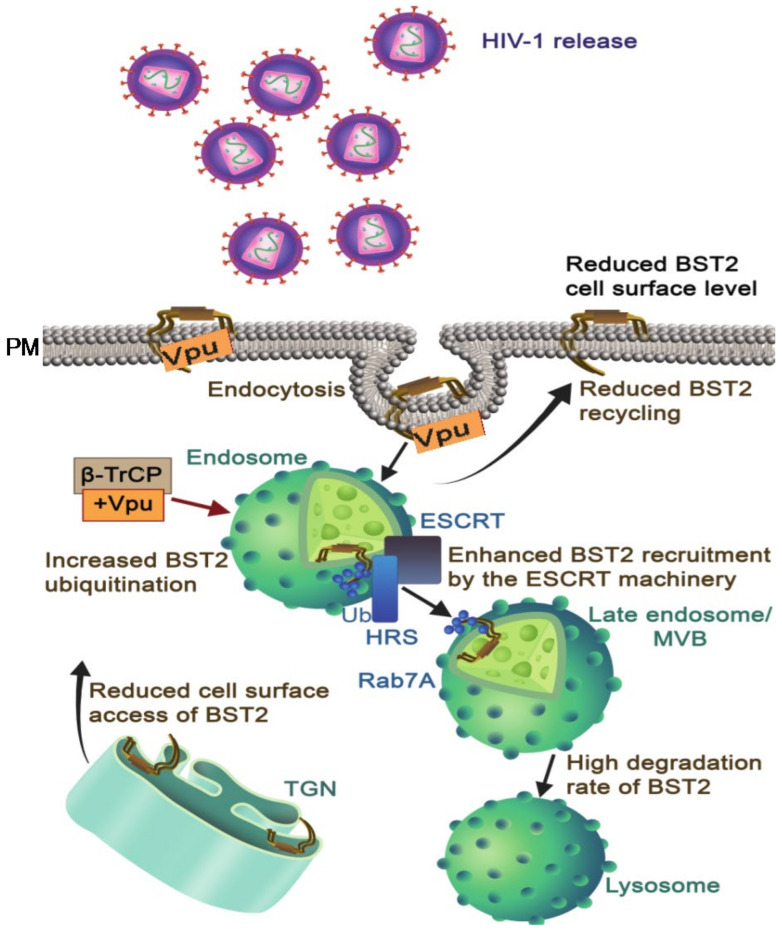
BST-2 surface downregulation and degradation by Vpu: In the absence of Vpu, BST-2 restricts newly synthesized viruses at the cell membrane, but Vpu downregulates BST-2 from the cell surface and promotes the release of virus particles from HIV-1-infected cells and enhances ubiquitination and lysosomal degradation of BST-2 by recruiting β-TrCP and exploiting the ESCRT pathway (ESCRT, HRS, and Rab7A). Besides, Vpu restricts BST-2 trafficking to the cell membrane from TGN and endosomes to reduce BST-2 level on the cell surface (indicates by arrows). Abbreviations: Vpu: viral protein U, BST-2: bone marrow stromal antigen 2, β-TrCP: β-transducin repeats-containing proteins, ESCRT: endosomal sorting complexes required for transport, Ub: ubiquitination, HRS: hepatocyte responsive serum phosphoprotein, MVB: multivesicular bodies, Rab7A, Ras-related protein 7A, TGN: trans-golgi network.

BST-2 is degraded by ubiquitination following recruitment of the SCF-β-TrCP-E3 ligase complex to the DS52GxxS56 motif that is present in the cytosolic region of Vpu [88,89] (Figure 3). Vpu enhances ubiquitination of BST-2 through lysine/serine and threonine amino acid residues present in the cytoplasmic tail of Vpu [89]. Vpu induces ubiquitination and degradation of both BST-2 and CD4 by identical molecular mechanisms, although the outcomes are different. Instead of targeting BST-2 to proteasomes, Vpu induces the β-TrCP-dependent sorting of BST-2 to lysosomes [77,84,88]. Moreover, it has been shown that the ESCRT complex and Rab7 are critical components of the endo-lysosomal trafficking involved in the degradation of BST-2 [90,91] (Figure 3).

Of the four HIV-1 groups, the M group is the most highly pathogenic and transmissible because Vpu is highly active, and this increases the ability of the virus to disseminate from one cell to another by counteracting the host protein BST-2 and evading the immune system (Table 1).

### 4.3. Immune Evasion to Virus Fitness and Survival

Innate immune responses play a significant role in host defenses against viral infections. Innate immune cells like natural killer (NK) and dendritic cells respond to invading viruses and contribute to controlling viral infection and replication during the initial stages of infection [92,93,94]. However, Vpu stabilizes HIV-1 infection and replication by evading immune responses by CD1d and NTB-A downmodulation [42,54]. Vpu also downregulates MHCII molecules from the cell surface to inhibit antigen presentation [39]. Together, these responses help HIV-1-infected cells escape cytotoxic and natural killer cells’ ability to kill the infected cell.

### 4.4. Cell Death

Vpu protein can be cytotoxic. It induces cell stress through induction of Fas ligand, p53 stabilization, and activating JNK signaling pathways [40,95,96,97]. Oxidative stress may also contribute to cell death because Vpu induces oxidative stress by stabilization of p53 protein, increasing TGF-β protein levels and increasing the release of cytotoxic substances from HIV-1-infected cells [40,98,99,100]. NADPH oxidase plays fundamental roles in the generation of reactive oxygen species (ROS) and can induce cell death [101,102,103]. P67phox, a subunit of NADPH oxidase enzyme, has TPR repeat sequences including the Vpu-interacting partner SGTA [104,105].

### 4.5. Regulation of Ion Channel Activity

Vpu can oligomerize its transmembrane domain and form pentamer ion channel pores selective for monovalent cations [16,24,26]. Vpu ion channel activity is regulated by serine (S23) amino acids that are conserved in HIV-1 M group viruses [106,107]. TASK-1, a mammalian two-pore potassium channel protein with structural homology protein to Vpu, stabilizes cell membrane potential [108,109]. Vpu interacts with TASK-1 proteins, inhibits its ion channel activity, and depolarizes plasma membranes to enhance cellular secretions [108,109,110].

### 4.6. Vpu Effects on HIV-1 LTR Activity

HIV-1 gene activation is dependent on host transcription factors including NF-κB, NFAT, and Ap-1 [111]. Vpu and its structural homolog TASK-1 inhibit transcription of unintegrated HIV-1 DNA in an NF-κB-dependent manner [112]. Vpu mutants (replaced transmembrane domain of Vpu with its structural homologs) also suppress virus production by reducing LTR activity by an unknown mechanism [112,113]. Thus, Vpu appears to be capable of regulating LTR activity to control virus production in infected cells possibly through the involvement of zinc finger proteins and histone deacetylase (HDAC) [114,115,116].

### 4.7. Vpu Effects on Endolysosomes

Vpu protein is localized to plasma membranes, ER, TGN complex, and endosomes [27,29]. Endosomes fuse with lysosomes and generate endolysosomes, which play crucial roles in physiological and pathological conditions such as antigen presentation, membrane trafficking, metabolism, autophagy, viral infections, cancer, neurological complications, and metabolic disorders [31,117,118,119,120,121,122,123,124,125,126]. Endolysosomes are highly acidic organelles and this acidity is regulated by the proton pump v-ATPase [127,128,129], BK channels [130], TRPML1 channels [130], and two-pore channels [131]. Vpu interacting protein ATP6V0C is a subunit of the v-ATPase pump and promotes intracellular aggregation of BST-2 and contributes to HIV-1 release [132]. However, much is still unclear about the effects of Vpu on v-ATPase, endolysosome acidification, endolysosomes’ regulatory functions, membrane trafficking, and autophagy.

Autophagy can enhance virus release and secretions from infected macrophage or monocyte cells [133,134,135]. HIV-1 enhances autophagy, while HIV-1 Nef blocks autophagy by direct interactions with Beclin and TFEB sequestration [136,137]. However, very little is currently known about the effects of Vpu on endolysosome degradation or autophagy pathways.

## 5. Conclusions

Vpu is an HIV-1 protein that counteracts host factors crucial for disseminating virus and disease progression. The primary targets of Vpu are cell surface host proteins that promote ubiquitination and proteasomal degradation processes [138,139,140]. Vpu might be targeted therapeutically to block the formation of heterooligomeric interactions between Vpu and host proteins at the cell surface as well as to suppress the progression of HIV-1 infection [141]. Moreover, Vpu disturbs the ubiquitination of host proteins by interacting with cellular factor β-TrCP through the cytosolic DSGxxS motif [138].

Hence, the transmembrane domain and DSxxSG motif in the cytosolic domain of Vpu may be targeted therapeutically against HIV-1 infection and disease progression.

## Figures and Tables

**Table 1:** Function of Vpu proteins of different HIV-1 groups.

Functions of Diverse Vpu Proteins				
Vpu functions	HIV-1 M	HIV-1 N	HIV-1 O	HIV-1 P
BST-2 downregulation [4,5,9,51,52,53]	Y	Y	N	N
CD4 degradation [4,5,9,51,52]	Y	N	Y	Y
NTB-A downmodulation [54,55]	Y	N	UN	UN
CD1d downmodulation [42,55,56]	Y	N	Y	Y
CCR7 downmodulation [43]	Y	UN	UN	UN

Abbreviations: Y: yes, N: no, UN: unknown, BST-2: bone marrow stromal antigen-2, NTB-A: natural killer T- and B-cell antigen, CD1d: cluster differentiation 1d, CCR7: CC-chemokine receptor-7, HIV-1 M: HIV-1 major, HIV-1 N: HIV-1 non-outlier, HIV-1 O: HIV-1 outlier, HIV-1 P: HIV-1 putative.

## Data Availability

Not applicable.

## Abbreviations

AIDS	Acquired immunodeficiency syndrome
AP-1	Activator protein 1
AP2	Adaptor complex 2
ATF4	Activating transcription factor 4
ATP6V0C	ATPase H^+^ transporting V0 subunit C
BAG6	BAG cochaperone 6
β-TrCP	Beta-transducin repeats-containing proteins
BK channel	Big-potassium channel
BST-2	Bone marrow stromal antigen 2
CCR7	CC-chemokine receptor 7
CD1d	Cluster differentiation 1d
CD317	Cluster of differentiation 317
CD4	Cluster of differentiation 4
CK-2	Casein kinase 2
COS	CV-1 (simian) in origin
ER	Endoplasmic reticulum
ERAD	Endoplasmic reticulum-associated degradation pathway
ESCRT	Endosomal sorting complexes required for transport
Gp120	Glycoprotein 120
Gp41	Glycoprotein 41
GPI	Glycosylphosphatidylinositol
HDAC	Histone deacetylase
HEK293T	Human embryonic kidney 293 cells containing SV40 T-antigen
HELA	Henrietta lacks cells
HIV-1	Human immunodeficiency virus 1
HIV-2	Human immunodeficiency virus 2
Hrs	Hepatocyte responsive serum phosphoprotein
IFN-α/β	Interferon-alpha/beta
JNK	Jun N-terminal kinase
LTR	Long terminal repeat
MHC I and MHC II	Major histocompatibility complex I and II
MVB	Multivesicular bodies
NADPH oxidase	Nicotinamide adenine dinucleotide phosphate oxidase
NBR1	Neighbor of BRCA1 gene 1
NDP52	Nuclear dot protein 52
Nef	Negative factor
NFAT	Nuclear factor of activated T-cells
NF-κB	Nuclear factor kappa light chain enhancer of activated B cells
NPL4	Nuclear protein localization protein 4
NTB-A	Natural killer T- and B-cell antigen
ORF	Open reading frame
p67-phox	P67 phagocyte oxidase
PM	Plasma membrane
Rab7	Ras-related protein 7
RNA	Ribonucleic acid
ROS	Reactive oxygen species
SCF	Skp1-Cullin-F-box protein
SIV cpz	Simian immunodeficiency viruses from chimpanzee
SIV	Simian immunodeficiency viruses
SIVagmTAN	Simian immunodeficiency virus from African green tantalus
monkeys	
SIVdent	Simian immunodeficiency virus from Dent’s mona monkey
SIVgor	Simian immunodeficiency virus from gorilla
SIVgs	Simian immunodeficiency virus from greater spot-nosed monkey
SIVmac	Simian immunodeficiency virus from rhesus macaques
SIVmon	Simian immunodeficiency virus from mona monkey
SIVsmm	Simian immunodeficiency virus from sooty mangabey
SGTA	Small glutamine-rich tetratricopeptide repeat
TASK-1	TWIK-related acid-sensitive K-1
TFEB	Transcription factor EB
TGF-β	Transforming growth factor-beta
TGN	Trans-Golgi network
TMD	Transmembrane domain
TPR	Tetratricopeptide repeat
TRPML-1	Transient receptor potential channel-1
Ub	Ubiquitination
UFD1L	Ubiquitin fusion degradation 1-like
V-ATPase	Vacuolar-type ATPase
VCP	Valosin-containing protein
Vpu	Viral protein U

## References

[1] Frankel A.D., Young J.A. (1998). HIV-1: Fifteen proteins and an RNA. Annu. Rev. Biochem..

[2] Engelman A., Cherepanov P. (2012). The structural biology of HIV-1: Mechanistic and therapeutic insights. Nat. Rev. Microbiol..

[3] Fanales-Belasio E., Raimondo M., Suligoi B., Buttò S. (2010). HIV virology and pathogenetic mechanisms of infection: A brief overview. Ann. Dell’istit. Super. Sanita.

[4] Sharp P.M., Hahn B.H. (2010). The evolution of HIV-1 and the origin of AIDS. Philos. Trans. R. Soc. Lond. B Biol. Sci..

[5] Sharp P.M., Hahn B.H. (2011). Origins of HIV and the AIDS pandemic. Cold Spring Harb. Perspect. Med..

[6] Strebel K., Klimkait T., Martin M.A. (1988). A novel gene of HIV-1, vpu, and its 16-kilodalton product. Science.

[7] Anderson J.L., Johnson A.T., Howard J.L., Purcell D.F. (2007). Both linear and discontinuous ribosome scanning are used for translation initiation from bicistronic human immunodeficiency virus type 1 env mRNAs. J. Virol..

[8] Schwartz S., Felber B., Pavlakis G. (1992). Mechanism of translation of monocistronic and multicistronic human immunodeficiency virus type 1 mRNAs. Mol. Cell Biol..

[9] Joas S., Parrish E.H., Gnanadurai C.W., Lump E., Stürzel C.M., Parrish N.F., Learn G.H., Sauermann U., Neumann B., Rensing K.M. (2018). Species-specific host factors rather than virus-intrinsic virulence determine primate lentiviral pathogenicity. Nat. Commun..

[10] Dazza M.C., Ekwalanga M., Nende M., Shamamba K.B., Bitshi P., Paraskevis D., Saragosti S. (2005). Characterization of a novel vpu-harboring simian immunodeficiency virus from a Dent’s Mona monkey (Cercopithecus mona denti). J. Virol..

[11] Courgnaud V., Abela B., Pourrut X., Mpoudi-Ngole E., Loul S., Delaporte E., Peeters M. (2003). Identification of a new simian immunodeficiency virus lineage with a vpu gene present among different cercopithecus monkeys (C. mona, C. cephus, and C. nictitans) from Cameroon. J. Virol..

[12] Barlow K., Ajao A., Clewley J. (2003). Characterization of a Novel Simian Immunodeficiency Virus (SIVmonNG1) Genome Sequence from a Mona Monkey (Cercopithecus mona). J. Virol..

[13] Kluge S.F., Sauter D., Vogl M., Peeters M., Li Y., Bibollet-Ruche F., Hahn B.H., Kirchhoff F. (2013). The transmembrane domain of HIV-1 Vpu is sufficient to confer anti-tetherin activity to SIVcpz and SIVgor Vpu proteins: Cytoplasmic determinants of Vpu function. Retrovirology.

[14] Strebel K., Klimkait T., Maldarelli F., Martin M.A. (1989). Molecular and biochemical analyses of human immunodeficiency virus type 1 vpu protein. J. Virol..

[15] Opella S., Park S., Lee S., Jones D., Nevzorov A., Mesleh M., Mrse A., Marassi F., Oblatt-Montal M., Montal M. (2005). Structure and Function of Vpu from HIV-1. Viral Membrane Proteins: Structure, Function, and Drug Design.

[16] Schubert U., Ferrer-Montiel A.V., Oblatt-Montal M., Henklein P., Strebel K., Montal M. (1996). Identification of an ion channel activity of the Vpu transmembrane domain and its involvement in the regulation of virus release from HIV-1-infected cells. FEBS Lett..

[17] Cohen E.A., Terwilliger E.F., Sodroski J.G., Haseltine W.A. (1988). Identification of a protein encoded by the vpu gene of HIV-1. Nature.

[18] Federau T., Schubert U., Flossdorf J., Henklein P., Schomburg D., Wray V. (1996). Solution structure of the cytoplasmic domain of the human immunodeficiency virus type 1 encoded virus protein U (Vpu). Int. J. Pept. Protein Res..

[19] González M.E. (2015). Vpu Protein: The Viroporin Encoded by HIV-1. Viruses.

[20] Bour S., Strebel K. (2003). The HIV-1 Vpu protein: A multifunctional enhancer of viral particle release. Microbes Infect..

[21] Wittlich M., Koenig B.W., Willbold D. (2008). Structural consequences of phosphorylation of two serine residues in the cytoplasmic domain of HIV-1 VpU. J. Pept. Sci. Off. Publ. Eur. Pept. Soc..

[22] Schubert U., Schneider T., Henklein P., Hoffmann K., Berthold E., Hauser H., Pauli G., Porstmann T. (1992). Human-immunodeficiency-virus-type-1-encoded Vpu protein is phosphorylated by casein kinase II. Eur. J. Biochem..

[23] Schubert U., Strebel K. (1994). Differential activities of the human immunodeficiency virus type 1-encoded Vpu protein are regulated by phosphorylation and occur in different cellular compartments. J. Virol..

[24] Ewart G.D., Sutherland T., Gage P.W., Cox G.B. (1996). The Vpu protein of human immunodeficiency virus type 1 forms cation-selective ion channels. J. Virol..

[25] Schubert U., Bour S., Ferrer-Montiel A.V., Montal M., Maldarell F., Strebel K. (1996). The two biological activities of human immunodeficiency virus type 1 Vpu protein involve two separable structural domains. J. Virol..

[26] Maldarelli F., Chen M.Y., Willey R.L., Strebel K. (1993). Human immunodeficiency virus type 1 Vpu protein is an oligomeric type I integral membrane protein. J. Virol..

[27] Pacyniak E., Gomez M.L., Gomez L.M., Mulcahy E.R., Jackson M., Hout D.R., Wisdom B.J., Stephens E.B. (2005). Identification of a region within the cytoplasmic domain of the subtype B Vpu protein of human immunodeficiency virus type 1 (HIV-1) that is responsible for retention in the golgi complex and its absence in the Vpu protein from a subtype C HIV-1. AIDS Res. Hum. Retrovir..

[28] Van Damme N., Goff D., Katsura C., Jorgenson R.L., Mitchell R., Johnson M.C., Stephens E.B., Guatelli J. (2008). The interferon-induced protein BST-2 restricts HIV-1 release and is downregulated from the cell surface by the viral Vpu protein. Cell Host Microbe.

[29] Ruiz A., Hill M.S., Schmitt K., Guatelli J., Stephens E.B. (2008). Requirements of the membrane proximal tyrosine and dileucine-based sorting signals for efficient transport of the subtype C Vpu protein to the plasma membrane and in virus release. Virology.

[30] Varthakavi V., Smith R.M., Martin K.L., Derdowski A., Lapierre L.A., Goldenring J.R., Spearman P. (2006). The pericentriolar recycling endosome plays a key role in Vpu-mediated enhancement of HIV-1 particle release. Traffic.

[31] Bonifacino J.S., Traub L.M. (2003). Signals for sorting of transmembrane proteins to endosomes and lysosomes. Annu. Rev. Biochem..

[32] Dubé M., Roy B.B., Guiot-Guillain P., Mercier J., Binette J., Leung G., Cohen É.A. (2009). Suppression of Tetherin-Restricting Activity upon Human Immunodeficiency Virus Type 1 Particle Release Correlates with Localization of Vpu in the *trans*-Golgi Network. J. Virol..

[33] Willey R.L., Maldarelli F., Martin M.A., Strebel K. (1992). Human immunodeficiency virus type 1 Vpu protein induces rapid degradation of CD4. J. Virol..

[34] Terwilliger E.F., Cohen E.A., Lu Y.C., Sodroski J.G., Haseltine W.A. (1989). Functional role of human immunodeficiency virus type 1 vpu. Proc. Natl. Acad. Sci. USA.

[35] Klimkait T., Strebel K., Hoggan M.D., Martin M.A., Orenstein J.M. (1990). The human immunodeficiency virus type 1-specific protein vpu is required for efficient virus maturation and release. J. Virol..

[36] Neil S.J., Zang T., Bieniasz P.D. (2008). Tetherin inhibits retrovirus release and is antagonized by HIV-1 Vpu. Nature.

[37] Sauter D., Specht A., Kirchhoff F. (2010). Tetherin: Holding on and letting go. Cell.

[38] Vincent M.J., Abdul Jabbar M. (1995). The human immunodeficiency virus type 1 Vpu protein: A potential regulator of proteolysis and protein transport in the mammalian secretory pathway. Virology.

[39] Hussain A., Wesley C., Khalid M., Chaudhry A., Jameel S. (2008). Human immunodeficiency virus type 1 Vpu protein interacts with CD74 and modulates major histocompatibility complex class II presentation. J. Virol..

[40] Verma S., Ali A., Arora S., Banerjea A.C. (2011). Inhibition of β-TrcP-dependent ubiquitination of p53 by HIV-1 Vpu promotes p53-mediated apoptosis in human T cells. Blood.

[41] Sandberg J.K., Andersson S.K., Bächle S.M., Nixon D.F., Moll M. (2012). HIV-1 Vpu interference with innate cell-mediated immune mechanisms. Curr. HIV Res..

[42] Moll M., Andersson S.K., Smed-Sörensen A., Sandberg J.K. (2010). Inhibition of lipid antigen presentation in dendritic cells by HIV-1 Vpu interference with CD1d recycling from endosomal compartments. Blood.

[43] Ramirez P.W., Famiglietti M., Sowrirajan B., DePaula-Silva A.B., Rodesch C., Barker E., Bosque A., Planelles V. (2014). Downmodulation of CCR7 by HIV-1 Vpu results in impaired migration and chemotactic signaling within CD4⁺ T cells. Cell Rep..

[44] Bour S., Geleziunas R., Wainberg M.A. (1995). The human immunodeficiency virus type 1 (HIV-1) CD4 receptor and its central role in promotion of HIV-1 infection. Microbiol. Rev..

[45] Ray N., Doms R.W. (2006). HIV-1 coreceptors and their inhibitors. Curr. Top. Microbiol. Immunol..

[46] Wildum S., Schindler M., Münch J., Kirchhoff F. (2006). Contribution of Vpu, Env, and Nef to CD4 down-modulation and resistance of human immunodeficiency virus type 1-infected T cells to superinfection. J. Virol..

[47] Willey R.L., Maldarelli F., Martin M.A., Strebel K. (1992). Human immunodeficiency virus type 1 Vpu protein regulates the formation of intracellular gp160-CD4 complexes. J. Virol..

[48] Buonocore L., Rose J.K. (1993). Blockade of human immunodeficiency virus type 1 production in CD4+ T cells by an intracellular CD4 expressed under control of the viral long terminal repeat. Proc. Natl. Acad. Sci. USA.

[49] Crise B., Buonocore L., Rose J.K. (1990). CD4 is retained in the endoplasmic reticulum by the human immunodeficiency virus type 1 glycoprotein precursor. J. Virol..

[50] Jabbar M.A., Nayak D.P. (1990). Intracellular interaction of human immunodeficiency virus type 1 (ARV-2) envelope glycoprotein gp160 with CD4 blocks the movement and maturation of CD4 to the plasma membrane. J. Virol..

[51] Sauter D., Schindler M., Specht A., Landford W.N., Münch J., Kim K.A., Votteler J., Schubert U., Bibollet-Ruche F., Keele B.F. (2009). Tetherin-driven adaptation of Vpu and Nef function and the evolution of pandemic and nonpandemic HIV-1 strains. Cell Host Microbe.

[52] Kirchhoff F. (2010). Immune Evasion and Counteraction of Restriction Factors by HIV-1 and Other Primate Lentiviruses. Cell Host Microbe.

[53] Sauter D., Hué S., Petit S.J., Plantier J.-C., Towers G.J., Kirchhoff F., Gupta R.K. (2011). HIV-1 Group P is unable to antagonize human tetherin by Vpu, Env or Nef. Retrovirology.

[54] Shah A.H., Sowrirajan B., Davis Z.B., Ward J.P., Campbell E.M., Planelles V., Barker E. (2010). Degranulation of natural killer cells following interaction with HIV-1-infected cells is hindered by downmodulation of NTB-A by Vpu. Cell Host Microbe.

[55] Sauter D., Unterweger D., Vogl M., Usmani S.M., Heigele A., Kluge S.F., Hermkes E., Moll M., Barker E., Peeters M. (2012). Human tetherin exerts strong selection pressure on the HIV-1 group N Vpu protein. PLoS Pathog..

[56] Bächle S.M., Sauter D., Sibitz S., Sandberg J.K., Kirchhoff F., Moll M. (2015). Involvement of a C-terminal motif in the interference of primate lentiviral Vpu proteins with CD1d-mediated antigen presentation. Sci. Rep..

[57] Magadán J.G., Bonifacino J.S. (2012). Transmembrane domain determinants of CD4 Downregulation by HIV-1 Vpu. J. Virol..

[58] Magadán J.G., Pérez-Victoria F.J., Sougrat R., Ye Y., Strebel K., Bonifacino J.S. (2010). Multilayered mechanism of CD4 downregulation by HIV-1 Vpu involving distinct ER retention and ERAD targeting steps. PLoS Pathog..

[59] Margottin F., Bour S.P., Durand H., Selig L., Benichou S., Richard V., Thomas D., Strebel K., Benarous R. (1998). A novel human WD protein, h-beta TrCp, that interacts with HIV-1 Vpu connects CD4 to the ER degradation pathway through an F-box motif. Mol. Cell.

[60] Evrard-Todeschi N., Gharbi-Benarous J., Bertho G., Coadou G., Megy S., Benarous R., Girault J.-P. (2006). NMR studies for identifying phosphopeptide ligands of the HIV-1 protein Vpu binding to the F-box protein β-TrCP. Peptides.

[61] Yao X.J., Garzon S., Boisvert F., Haseltine W.A., Cohen E.A. (1993). The effect of vpu on HIV-1-induced syncytia formation. J. Acquir. Immune Defic. Syndr..

[62] Schubert U., Clouse K.A., Strebel K. (1995). Augmentation of virus secretion by the human immunodeficiency virus type 1 Vpu protein is cell type independent and occurs in cultured human primary macrophages and lymphocytes. J. Virol..

[63] Sakai H., Tokunaga K., Kawamura M., Adachi A. (1995). Function of human immunodeficiency virus type 1 Vpu protein in various cell types. J. Gen. Virol..

[64] Geraghty R.J., Talbot K.J., Callahan M., Harper W., Panganiban A.T. (1994). Cell type-dependence for Vpu function. J. Med. Primatol..

[65] Neil S.J. (2013). The antiviral activities of tetherin. Curr. Top. Microbiol. Immunol..

[66] Le Tortorec A., Willey S., Neil S.J.D. (2011). Antiviral inhibition of enveloped virus release by tetherin/BST-2: Action and counteraction. Viruses.

[67] Jia B., Serra-Moreno R., Neidermyer W., Rahmberg A., Mackey J., Fofana I.B., Johnson W.E., Westmoreland S., Evans D.T. (2009). Species-specific activity of SIV Nef and HIV-1 Vpu in overcoming restriction by tetherin/BST2. PLoS Pathog..

[68] Zhang F., Wilson S.J., Landford W.C., Virgen B., Gregory D., Johnson M.C., Munch J., Kirchhoff F., Bieniasz P.D., Hatziioannou T. (2009). Nef proteins from simian immunodeficiency viruses are tetherin antagonists. Cell Host Microbe.

[69] Serra-Moreno R., Jia B., Breed M., Alvarez X., Evans D.T. (2011). Compensatory changes in the cytoplasmic tail of gp41 confer resistance to tetherin/BST-2 in a pathogenic nef-deleted SIV. Cell Host Microbe.

[70] Le Tortorec A., Neil S.J. (2009). Antagonism to and intracellular sequestration of human tetherin by the human immunodeficiency virus type 2 envelope glycoprotein. J. Virol..

[71] Hauser H., Lopez L.A., Yang S.J., Oldenburg J.E., Exline C.M., Guatelli J.C., Cannon P.M. (2010). HIV-1 Vpu and HIV-2 Env counteract BST-2/tetherin by sequestration in a perinuclear compartment. Retrovirology.

[72] Gupta R.K., Mlcochova P., Pelchen-Matthews A., Petit S.J., Mattiuzzo G., Pillay D., Takeuchi Y., Marsh M., Towers G.J. (2009). Simian immunodeficiency virus envelope glycoprotein counteracts tetherin/BST-2/CD317 by intracellular sequestration. Proc. Natl. Acad. Sci. USA.

[73] Kupzig S., Korolchuk V., Rollason R., Sugden A., Wilde A., Banting G. (2003). Bst-2/HM1.24 is a raft-associated apical membrane protein with an unusual topology. Traffic.

[74] Masuyama N., Kuronita T., Tanaka R., Muto T., Hirota Y., Takigawa A., Fujita H., Aso Y., Amano J., Tanaka Y. (2009). HM1.24 is internalized from lipid rafts by clathrin-mediated endocytosis through interaction with alpha-adaptin. J. Biol. Chem..

[75] Perez-Caballero D., Zang T., Ebrahimi A., McNatt M.W., Gregory D.A., Johnson M.C., Bieniasz P.D. (2009). Tetherin inhibits HIV-1 release by directly tethering virions to cells. Cell.

[76] Hammonds J., Wang J.-J., Yi H., Spearman P. (2010). Immunoelectron microscopic evidence for Tetherin/BST2 as the physical bridge between HIV-1 virions and the plasma membrane. PLoS Pathog..

[77] Iwabu Y., Fujita H., Kinomoto M., Kaneko K., Ishizaka Y., Tanaka Y., Sata T., Tokunaga K. (2009). HIV-1 accessory protein Vpu internalizes cell-surface BST-2/tetherin through transmembrane interactions leading to lysosomes. J. Biol. Chem..

[78] Kobayashi T., Ode H., Yoshida T., Sato K., Gee P., Yamamoto S.P., Ebina H., Strebel K., Sato H., Koyanagi Y. (2011). Identification of amino acids in the human tetherin transmembrane domain responsible for HIV-1 Vpu interaction and susceptibility. J. Virol..

[79] Vigan R., Neil S.J.D. (2010). Determinants of Tetherin Antagonism in the Transmembrane Domain of the Human Immunodeficiency Virus Type 1 Vpu Protein. J. Virol..

[80] Skasko M., Wang Y., Tian Y., Tokarev A., Munguia J., Ruiz A., Stephens E.B., Opella S.J., Guatelli J. (2012). HIV-1 Vpu protein antagonizes innate restriction factor BST-2 via lipid-embedded helix-helix interactions. J. Biol. Chem..

[81] Pickering S., Hué S., Kim E.Y., Reddy S., Wolinsky S.M., Neil S.J. (2014). Preservation of tetherin and CD4 counter-activities in circulating Vpu alleles despite extensive sequence variation within HIV-1 infected individuals. PLoS Pathog..

[82] McNatt M.W., Zang T., Bieniasz P.D. (2013). Vpu binds directly to tetherin and displaces it from nascent virions. PLoS Pathog..

[83] Rollason R., Korolchuk V., Hamilton C., Schu P., Banting G. (2007). Clathrin-mediated endocytosis of a lipid-raft-associated protein is mediated through a dual tyrosine motif. J. Cell Sci..

[84] Mitchell R.S., Katsura C., Skasko M.A., Fitzpatrick K., Lau D., Ruiz A., Stephens E.B., Margottin-Goguet F., Benarous R., Guatelli J.C. (2009). Vpu antagonizes BST-2-mediated restriction of HIV-1 release via beta-TrCP and endo-lysosomal trafficking. PLoS Pathog..

[85] Schmidt S., Fritz J.V., Bitzegeio J., Fackler O.T., Keppler O.T. (2011). HIV-1 Vpu Blocks Recycling and Biosynthetic Transport of the Intrinsic Immunity Factor CD317/Tetherin To Overcome the Virion Release Restriction. mBio.

[86] Lau D., Kwan W., Guatelli J. (2011). Role of the endocytic pathway in the counteraction of BST-2 by human lentiviral pathogens. J. Virol..

[87] Dubé M., Paquay C., Roy B.B., Bego M.G., Mercier J., Cohen E.A. (2011). HIV-1 Vpu antagonizes BST-2 by interfering mainly with the trafficking of newly synthesized BST-2 to the cell surface. Traffic.

[88] Douglas J.L., Viswanathan K., McCarroll M.N., Gustin J.K., Früh K., Moses A.V. (2009). Vpu directs the degradation of the human immunodeficiency virus restriction factor BST-2/Tetherin via a {beta}TrCP-dependent mechanism. J. Virol..

[89] Tokarev A.A., Munguia J., Guatelli J.C. (2011). Serine-threonine ubiquitination mediates downregulation of BST-2/tetherin and relief of restricted virion release by HIV-1 Vpu. J. Virol..

[90] Janvier K., Pelchen-Matthews A., Renaud J.B., Caillet M., Marsh M., Berlioz-Torrent C. (2011). The ESCRT-0 component HRS is required for HIV-1 Vpu-mediated BST-2/tetherin down-regulation. PLoS Pathog..

[91] Caillet M., Janvier K., Pelchen-Matthews A., Delcroix-Genête D., Camus G., Marsh M., Berlioz-Torrent C. (2011). Rab7A is required for efficient production of infectious HIV-1. PLoS Pathog..

[92] Tupin E., Kinjo Y., Kronenberg M. (2007). The unique role of natural killer T cells in the response to microorganisms. Nat. Rev. Microbiol..

[93] Mattner J., Debord K.L., Ismail N., Goff R.D., Cantu C., Zhou D., Saint-Mezard P., Wang V., Gao Y., Yin N. (2005). Exogenous and endogenous glycolipid antigens activate NKT cells during microbial infections. Nature.

[94] Altfeld M., Gale M. (2015). Innate immunity against HIV-1 infection. Nat. Immunol..

[95] Casella C.R., Rapaport E.L., Finkel T.H. (1999). Vpu Increases Susceptibility of Human Immunodeficiency Virus Type 1-Infected Cells to Fas Killing. J. Virol..

[96] Akari H., Bour S., Kao S., Adachi A., Strebel K. (2001). The human immunodeficiency virus type 1 accessory protein Vpu induces apoptosis by suppressing the nuclear factor kappaB-dependent expression of antiapoptotic factors. J. Exp. Med..

[97] Marchal C., Vinatier G., Sanial M., Plessis A., Pret A.M., Limbourg-Bouchon B., Théodore L., Netter S. (2012). The HIV-1 Vpu protein induces apoptosis in Drosophila via activation of JNK signaling. PLoS ONE.

[98] Patel P., Khan N., Rani M., Gupta D., Jameel S. (2014). The expression of HIV-1 Vpu in monocytes causes increased secretion of TGF-β that activates profibrogenic genes in hepatic stellate cells. PLoS ONE.

[99] Niwa-Kawakita M., Ferhi O., Soilihi H., Le Bras M., Lallemand-Breitenbach V., de Thé H. (2017). PML is a ROS sensor activating p53 upon oxidative stress. J. Exp. Med..

[100] Liu R.-M., Desai L.P. (2015). Reciprocal regulation of TGF-β and reactive oxygen species: A perverse cycle for fibrosis. Redox Biol..

[101] Tarafdar A., Pula G. (2018). The Role of NADPH Oxidases and Oxidative Stress in Neurodegenerative Disorders. Int. J. Mol. Sci..

[102] Coyoy A., Valencia A., Guemez-Gamboa A., Morán J. (2008). Role of NADPH oxidase in the apoptotic death of cultured cerebellar granule neurons. Free Radic. Biol. Med..

[103] Babior B.M. (2004). NADPH oxidase. Curr. Opin. Immunol..

[104] Dutta S., Tan Y.-J. (2008). Structural and Functional Characterization of Human SGT and Its Interaction with Vpu of the Human Immunodeficiency Virus Type 1. Biochemistry.

[105] Lapouge K., Smith S.J., Walker P.A., Gamblin S.J., Smerdon S.J., Rittinger K. (2000). Structure of the TPR domain of p67phox in complex with Rac.GTP. Mol. Cell.

[106] Padhi S., Khan N., Jameel S., Priyakumar U.D. (2013). Molecular dynamics simulations reveal the HIV-1 Vpu transmembrane protein to form stable pentamers. PLoS ONE.

[107] Mehnert T., Routh A., Judge P.J., Lam Y.H., Fischer D., Watts A., Fischer W.B. (2008). Biophysical characterization of Vpu from HIV-1 suggests a channel-pore dualism. Proteins.

[108] Hsu K., Seharaseyon J., Dong P., Bour S., Marbán E. (2004). Mutual functional destruction of HIV-1 Vpu and host TASK-1 channel. Mol. Cell.

[109] Strebel K. (2004). HIV-1 Vpu: Putting a Channel to the TASK. Mol. Cell.

[110] Hsu K., Han J., Shinlapawittayatorn K., Deschenes I., Marbán E. (2010). Membrane Potential Depolarization as a Triggering Mechanism for Vpu-Mediated HIV-1 Release. Biophys. J..

[111] Kilareski E.M., Shah S., Nonnemacher M.R., Wigdahl B. (2009). Regulation of HIV-1 transcription in cells of the monocyte-macrophage lineage. Retrovirology.

[112] Emeagwali N., Hildreth J.E. (2012). Human immunodeficiency virus type 1 Vpu and cellular TASK proteins suppress transcription of unintegrated HIV-1 DNA. Virol. J..

[113] Khan N., Padhi S., Patel P., Priyakumar U.D., Jameel S. (2020). The HIV-1 Vpu transmembrane domain topology and formation of a hydrophobic interface with BST-2 are critical for Vpu-mediated BST-2 downregulation. bioRxiv.

[114] Keedy K.S., Archin N.M., Gates A.T., Espeseth A., Hazuda D.J., Margolis D.M. (2009). A limited group of class I histone deacetylases acts to repress human immunodeficiency virus type 1 expression. J. Virol..

[115] Reynolds L., Ullman C., Moore M., Isalan M., West M.J., Clapham P., Klug A., Choo Y. (2003). Repression of the HIV-1 5’ LTR promoter and inhibition of HIV-1 replication by using engineered zinc-finger transcription factors. Proc. Natl. Acad. Sci. USA.

[116] Nishitsuji H., Abe M., Sawada R., Takaku H. (2012). ZBRK1 represses HIV-1 LTR-mediated transcription. FEBS Lett..

[117] Bright N.A., Davis L.J., Luzio J.P. (2016). Endolysosomes Are the Principal Intracellular Sites of Acid Hydrolase Activity. Curr. Biol..

[118] Bright N.A., Gratian M.J., Luzio J.P. (2005). Endocytic Delivery to Lysosomes Mediated by Concurrent Fusion and Kissing Events in Living Cells. Curr. Biol..

[119] Chen X., Wagener J.F., Morgan D.H., Hui L., Ghribi O., Geiger J.D. (2010). Endolysosome mechanisms associated with Alzheimer’s disease-like pathology in rabbits ingesting cholesterol-enriched diet. J. Alzheimers Dis..

[120] Huotari J., Helenius A. (2011). Endosome maturation. EMBO J..

[121] Perera R.M., Zoncu R. (2016). The Lysosome as a Regulatory Hub. Annu. Rev. Cell Dev. Biol..

[122] Khan N., Chen X., Geiger J.D. (2020). Role of Endolysosomes in Severe Acute Respiratory Syndrome Coronavirus-2 Infection and Coronavirus Disease 2019 Pathogenesis: Implications for Potential Treatments. Front. Pharm..

[123] Khan N., Haughey N.J., Nath A., Geiger J.D. (2019). Involvement of organelles and inter-organellar signaling in the pathogenesis of HIV-1 associated neurocognitive disorder and Alzheimer’s disease. Brain Res..

[124] Khan N.C.X., Geiger J.D. (2021). Possible Therapeutic Use of Natural Compounds Against COVID-19. J. Cell Signal.

[125] Truschel S.T., Clayton D.R., Beckel J.M., Yabes J.G., Yao Y., Wolf-Johnston A., Birder L.A., Apodaca G. (2018). Age-related endolysosome dysfunction in the rat urothelium. PLoS ONE.

[126] Munz C. (2012). Antigen Processing for MHC Class II Presentation via Autophagy. Front. Immunol..

[127] Colacurcio D.J., Nixon R.A. (2016). Disorders of lysosomal acidification—The emerging role of v-ATPase in aging and neurodegenerative disease. Ageing Res. Rev..

[128] Mindell J.A. (2012). Lysosomal acidification mechanisms. Annu. Rev. Physiol..

[129] Collins M.P., Forgac M. (2018). Regulation of V-ATPase Assembly in Nutrient Sensing and Function of V-ATPases in Breast Cancer Metastasis. Front. Physiol..

[130] Khan N., Lakpa K.L., Halcrow P.W., Afghah Z., Miller N.M., Geiger J.D., Chen X. (2019). BK channels regulate extracellular Tat-mediated HIV-1 LTR transactivation. Sci. Rep..

[131] Khan N., Halcrow P.W., Lakpa K.L., Afghah Z., Miller N.M., Dowdy S.F., Geiger J.D., Chen X. (2020). Two-pore channels regulate Tat endolysosome escape and Tat-mediated HIV-1 LTR transactivation. FASEB J. Off. Publ. Fed. Am. Soc. Exp. Biol..

[132] Waheed A.A., Swiderski M., Khan A., Gitzen A., Majadly A., Freed E.O. (2020). The viral protein U (Vpu)-interacting host protein ATP6V0C down-regulates cell-surface expression of tetherin and thereby contributes to HIV-1 release. J. Biol. Chem..

[133] Killian M.S. (2012). Dual role of autophagy in HIV-1 replication and pathogenesis. AIDS Res. Ther..

[134] Kyei G.B., Dinkins C., Davis A.S., Roberts E., Singh S.B., Dong C., Wu L., Kominami E., Ueno T., Yamamoto A. (2009). Autophagy pathway intersects with HIV-1 biosynthesis and regulates viral yields in macrophages. J. Cell Biol..

[135] Borel S., Espert L., Biard-Piechaczyk M. (2012). Macroautophagy Regulation during HIV-1 Infection of CD4+ T Cells and Macrophages. Front. Immunol..

[136] Campbell G.R., Rawat P., Bruckman R.S., Spector S.A. (2015). Human Immunodeficiency Virus Type 1 Nef Inhibits Autophagy through Transcription Factor EB Sequestration. PLoS Pathog..

[137] Castro-Gonzalez S., Shi Y., Colomer-Lluch M., Song Y., Mowery K., Almodovar S., Bansal A., Kirchhoff F., Sparrer K., Liang C. (2021). HIV-1 Nef counteracts autophagy restriction by enhancing the association between BECN1 and its inhibitor BCL2 in a PRKN-dependent manner. Autophagy.

[138] Dubé M., Bego M.G., Paquay C., Cohen É.A. (2010). Modulation of HIV-1-host interaction: Role of the Vpu accessory protein. Retrovirology.

[139] Prévost J., Edgar C.R., Richard J., Trothen S.M., Jacob R.A., Mumby M.J., Pickering S., Dubé M., Kaufmann D.E., Kirchhoff F. (2020). HIV-1 Vpu Downregulates Tim-3 from the Surface of Infected CD4^+^ T Cells. J. Virol..

[140] Bolduan S., Reif T., Schindler M., Schubert U. (2014). HIV-1 Vpu mediated downregulation of CD155 requires alanine residues 10, 14 and 18 of the transmembrane domain. Virology.

[141] Montal M. (2009). Vpu matchmakers as a therapeutic strategy for HIV infection. PLoS Pathog..

